# Evaluation of Matrix Issues in the Applicability of the Neuro-2a Cell Based Assay on the Detection of CTX in Fish Samples

**DOI:** 10.3390/toxins12050308

**Published:** 2020-05-09

**Authors:** David Castro, Ronald Manger, Oscar Vilariño, Ana Gago-Martínez

**Affiliations:** 1 Biomedical Research Center (CINBIO), Department of Analytical and Food Chemistry, University of Vigo, Campus Universitario de Vigo, 36310 Vigo, Spain; dcastro@uvigo.es (D.C.); ovilarino@uvigo.es (O.V.); 2 Fred Hutchinson Cancer Research Center (retired), Seattle, WA 98109, USA; rlmanger@yahoo.com

**Keywords:** ciguatoxins, neuroblastoma cell assay, matrix effect

## Abstract

Ciguatoxins (CTXs) are a group of neurotoxins responsible for the syndrome ciguatera fish poisoning (CFP) as a result of the consumption of contaminated fish. The presence of these toxins has been detected around the Pacific, Caribbean and Indian coasts. Recent reports indicate the emergence of CFP in other geographic areas, in particular in European coasts, of the Canary Islands (Spain) and Madeira (Portugal). A neuroblastoma cell line of murine origin (N2a) has been applied to assay different groups of neurotoxins, acting on voltage-gated sodium channel (VGSC) of excitable cells, N2a-MTT. The great potential of N2a-MTT as a sensitive tool for the CTXs screening is clearly recognized, notably because it allows the detection of these toxins at levels below recommended as security levels. However, the complexity of the matrix is a critical point on the application of N2a-MTT, which needs to be evaluated. The aim of this work is to provide recommendations for an implemented N2a-MTT method for CTXs determination in fish that avoids matrix effects, particularly those related to high lipid content.

## 1. Introduction

Ciguatera poisoning (CP) is a non-bacterial food intoxication endemic in tropical and subtropical regions of the world and caused by the consumption of fish contaminated with ciguatoxins (CTXs) [1,2]. CTXs are ladder-like cyclic polyethers, lipophilic and are stable to pH and temperature; which are produced by fish metabolization of its algal precursors [3,4]. There is uncertainty about their algal precursors but different benthic dinoflagellates such as *Gambierdiscus* spp. [5,6,7] or *Fukuyoa* spp. [8] were identified as producers of CTX-like compounds [9,10]. CTXs are classified depending on the geographical region they appear as Pacific, Indian and Caribbean Ciguatoxins (P-CTXs, I-CTXs and C-CTXs) [11]. P-CTXs are widely distributed in the Pacific and some regions of the Indian Ocean, I-CTXs are not yet elucidated and seem to be present in the Indian Ocean and C-CTXs are detected in fish from the Caribbean Sea and have recently emerged in Macaronesia (Northeast Atlantic), specifically in the Canary Islands (Spain) and Madeira (Portugal) [12,13,14].

The US Food and Drug Administration (FDA) established a guidance level of 0.01 ng/g fish tissue for the most potent congener CTX1B and 0.1 ng/g for C-CTX1 [15]. On the other hand, there are no regulatory limits in Europe, where the European Food Safety Authority (EFSA) published a scientific opinion about CTXs, where they highlighted the importance of developing analytical methods for CTXs evaluation, recommending in vitro assays for screening and liquid chromatography coupled with tandem mass spectrometry (LC–MS/MS) for confirmation [16].

Different strategies for in vitro assays have been developed for the monitoring purpose of CTXs, including pharmacological (i.e., receptor binding assay) [17,18,19], cytotoxicity [20,21,22,23,24,25], immunochemical approaches (ELISA) [26,27,28] or reporter gene assays [29,30]. The in vitro assay most widely applied in the CTXs screening is a cytotoxicity cell based assay using a neuroblastoma cell line of murine origin (N2a) and measuring the mitochondrial activity of viable cells by MTT colorimetric assay (N2a-MTT). N2a-MTT was originally developed by Manger et al. [21,22] and it is based on the CTXs mechanism of action in the voltage-gated sodium channels (VGSC) of excitable cells [17,18,31,32]. The specificity towards the effects of certain VGSC toxins, such as CTXs, is achieved by the pretreatment of cells with veratridine (V) and ouabain (O), cells untreated with both compounds are not sensitive to these types of specific toxins. Additionally, despite not being specific for CTXs alone, this procedure allows to distinguish between sodium channel-enhancing toxins, also called CTX-like compounds such as ciguatoxins and brevetoxins (PbTxs) and blocking toxins such as saxitoxin (STXs) and tetrodotoxin (TTXs) [33,34]. The main advantage compared to the traditional mouse bioassay (MBA), apart from animal welfare issues, is its higher sensitivity and ability to meet the US FDA guidance level.

Its importance is also found in the capacity of qualitatively and semi quantitatively estimating the total presence of CTX-like compounds in contaminated samples. Due to the lack of commercially available standards and reference materials its combination with sample fractionation and LC–MS/MS it is a useful approach in the identification of CTXs analogues.

Although N2a-MTT is a very sensitive tool to monitor CTX-like compounds its main disadvantage is that the specificity can be influenced by possible matrix effects contributing to non-specific toxic effects, interfering with CTXs detection and reducing the reliability of the assay. Therefore, since the exposure to excessive amount of matrix compounds (i.e., lipids) may be toxic to N2a cells, it is necessary to establish a maximum tissue dose equivalent (MTDE) [35]. However, the MTDE can potentially vary according to different characteristics such as fish species, origin and season of capture. N2a-MTT has been used for the discrimination of CTXs in a wide variety of biological matrices (i.e., dinoflagellates and blood, mussels) [35,36,37,38]. However, a few studies have documented the relationship between non-specific toxic effects in N2a and fish lipid content. The study by Caillaud et al. (2012) [35] is the first report that provided information about the maximal concentration of fish tissue equivalent that did not induce non-specific toxic effects in the analysis of species of amberjack and wahoo harvested in the Macaronesian region. However, this study did not provide information regarding interference of the matrix associated with different species or other factors. To help address these issues, previous studies highlighted the importance of using efficient sample pretreatment protocols as an important factor in modulating matrix interference [22,35,36,37,38].

The objective of this work was the evaluation and elimination of interfering matrix effects on the N2a-MTT assay, thereby contributing to an increased sensitivity and reliability of the method. For these studies several fish species widely consumed in the Canary Islands where this study was focused, were selected. The species selected also took into account an important nutritional parameter, fat content, as a potentially significant component for the observed matrix effect in N2a-MTT assays. The methodology and results obtained from this evaluation facilitate analysis of the MTDE required to avoid non-specific toxic effects and increase both sensitivity and reliability of this cell-based assay.

For VGSC-activating toxins, the reduction on cell viability in the presence of ouabain and veratridine (OV) shows a typical sigmoidal dose-response curve. Consequently, the effects of the matrix on CTXs analogues toxicity were also evaluated to exclude a possible response of interfering endogenous compounds on VGSCs.

## 2. Results and Discussion

To our knowledge this is the first study where the matrix effect of different fish species from Macaronesia (Eastern Atlantic) were evaluated with the objective of minimizing non-specific toxic effects. The control of these non-specific toxic effects would facilitate the reduction in the number of false positives or negatives and therefore increase the reliability of the assay.

Five different uncontaminated fish species prone to contain CTXs were extracted under the conditions described by (Estevez et al. 2019a) [39] and the non-specific toxic effects were evaluated in non-purified and purified by solid phase extraction (SPE) extracts (Table 1).

Although there is not an established official classification, fish species used in this work were grouped depending on their total lipid content (% of lipid), being NC1 and NC2 considered as low-fat fish (<3% of lipid), NC3 and NC4 as semi-fat fish (between 3% and 6% of lipid) and NC5 as fat fish (>6% lipid content)

### 2.1. Matrix Effect in Non-Purified Sample Extracts

To determine matrix effects alone, non-purified fish extracts were analyzed without the addition of OV compounds. Non-purified fish extracts were analyzed at two equivalent tissue (TE) doses, 25 and 2.5 mg TE, in order to demonstrate the relation between lipid fish content and non-specific toxic effects (cell viability ≥ 90% of control).

**Figure 1:**
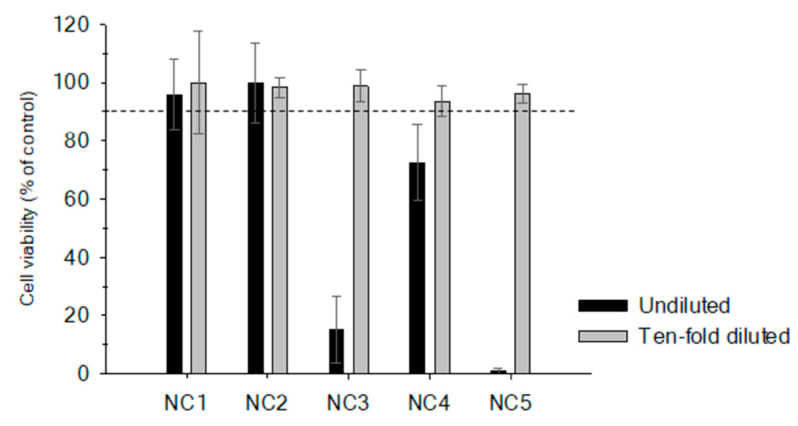
Cell viability after 16 h exposure to ciguatoxin (CTX)-negative fish extracts without the clean-up step: *Pagrus pagrus* (NC1); *Epinephelus marginatus* (NC2); *Pomatomus saltatrix* (NC3); *Seriola dumerili* (NC4) and *Acantocybium solandri* (NC5). Black bars show cell viability for a tissue equivalent dose of 25 mg and gray bars represent the ten-fold diluted fish extracts (2.5 mg two equivalent tissue (TE) dose). Dotted line represents 90% of cell viability, limit of non-specific toxic effects. Each extract was analyzed in triplicate and error bars represent assay variability.

When 25 mg TE of these fish extracts were added, NC1 and NC2 did not exhibit any cytotoxic effect whereas NC3, NC4 and NC5 showed a cytotoxic effect reducing cell viability to levels below 20%. Results show that the cytotoxic effects increase with the total fatty acids content and a 10-fold dilution (2.5 mg TE) was needed to avoid these non-specific toxic effects (Figure 1). Although sample dilution is a well-known alternative to reduce the matrix effect, this approach endangers the detection of potential CTX-like compounds when the sample has a low contamination.

**Figure 2:**
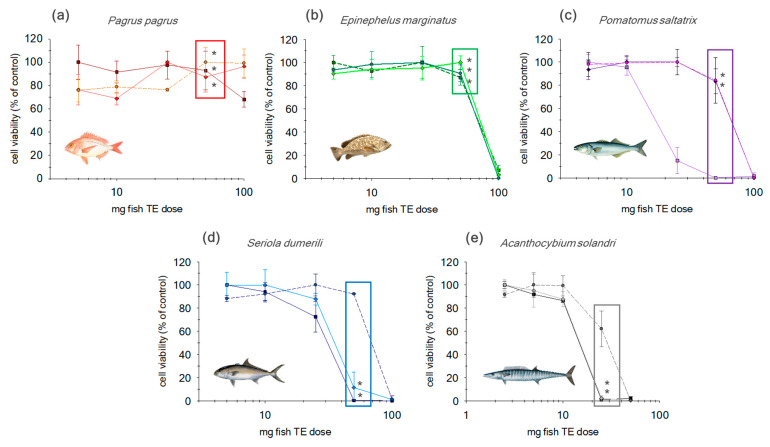
Viability assay in N2a cells exposed for 16 h to CTX-negative fish extracts obtained by different steps of purification for **(a)** *Pagrus pagrus* (NC1); **(b)** *Epinephelus marginatus* (NC2); **(c)** *Pomatomus saltatrix* (NC3); **(d)** *Seriola dumerili* (NC4) and (e) *Acantocybium solandri* (NC5) by: ■ No cleanup step, ♦ SPE-Florisil and ● SPE-Florisil + C18. Data are expressed as mean ± SD (*n* = 3). The asterisks (boxed area) show homogenous groups when 50 mg of tissue equivalent was added (ANOVA, Bonferroni’s HSD test, *p* < 0.05).

Additionally, non-purified extracts were analyzed in a working range from 0.1 TE to 100 mg. At a dose > 50 mg TE, non-specific toxic effects can be observed for the low-fat fish (NC1 and NC2) (Figure 2). However, for semi- and fat fish (NC3, NC4 and NC5) these effects were observed for amounts > 10 mg TE. Consequently, in order to minimize matrix effect without compromise to sensitivity two alternatives were proposed: 1) incorporation of additional clean up step or 2) establishing a maximum tissue dose equivalent (MTDE) for different species.

### 2.2. Evaluation of Additional Cleanup Steps

Previous studies highlighted the importance of using different clean-up procedures to selectively remove matrix interferences and increase the specificity of the assays [35,40,41,42,43,44]. Prior work carried out by the research team involved in this study was focused on the implementation of sample pretreatment to improve performance of the LC–MS/MS method for the analysis of CTXs (Estevez et al. 2019) [39]. In addition to optimization of the LC–MS/MS conditions, improvements of the sample pretreatment were performed, in particular the purification step, to efficiently remove matrix effects and interfering compounds. The optimized sample pretreatment protocol includes a cleanup in two steps by solid phase extraction (SPE) with two different separation mechanisms and was the same used in the present study. As detailed in the previous study, the efficiency of these SPE steps has been evaluated in terms of overall CTX recovery of 72.7% [39].

#### 2.2.1. SPE-Florisil

Purified fish extracts through SPE-Florisil were analyzed in a working range from 0.1 TE to 100 mg (Figure 2). Results showed that N2a cells did not exhibit non-specific toxic effects throughout the working range in NC1 fish extracts. At a dose > 50 mg TE, non-specific toxic effects can be observed for the low-fat fish (NC2). At a dose of 50 mg of TE of the lower lipid content fish species no significant differences in cell viability were observed compared to the non-purified extracts. For the semi-fat fish group, NC3 showed a similar response to the low-fat fish with non-specific toxic effects for tissue amounts > 50 mg. Contrary statistically significant differences were observed compared to extracts without cleanup for NC3 and NC4. For NC4, non-specific toxic effects were observed when 50 mg TE were added to the assay, with 25 mg TE being the adequate amount to avoid significant matrix effect. SPE Florisil showed a significate but minor improvement (around 15%) in the reduction of non-specific toxic effects for this particular fish species. Finally, for NC5 (fat fish) extracts, at 50 mg TE an important reduction on cell viability was observed for non-purified and purified extracts. Therefore, a maximum dose of 10 mg TE must be added in order to avoid non-specific toxic effects. Results show no significant effect over cell viability when a SPE-Florisil was applied.

#### 2.2.2. SPE-Florisil + C18

An additional SPE C18 step was evaluated in the same working range (0.1–100 mg TE; Figure 2). NC1 and NC2 samples did not manifest any significant difference at 50 mg TE. Therefore, the introduction of an additional step of SPE C18 did not offer any improvement compared to SPE-Florisil.

A different performance was observed in NC3 and NC4 (semi-fat fish) after the incorporation of SPE-C18. By selecting 50 mg TE no significant effect was observed for NC3 compared to the SPE Florisil, showing non-specific toxic effects at a dose > 50 mg TE. In contrasts, in NC4 extract, the incorporation of an additional SPE-C18 allowed for an increase of the maximum dose from 25 to 50 mg TE.

Finally, in NC5 extracts, despite an observed significant reduction in matrix effect after SPE-C18 (35%) at 25 mg TE, this step did not adequately improve the interference removal.

As observed the efficiency of the different SPE treatments depended on the lipid content of the fish species. The low-fat fish (NC1 and NC2) slightly increased the maximum tissue dose equivalent, MTDE, on a 26% basis. When NC3 was analyzed incorporation of SPE-C18 offered no significant improvement with results obtained after the SPE-Florisil step alone. Nevertheless, the statistically significant differences between three treatments shows that employing both purification steps worked very well in order to remove non-specific toxic effects. NC5 (fat fish) was also considerably affected by the addition of further clean-up. In this case, incorporation of additional cleanup steps, including SPE C18, did not eliminate non-specific toxic effects when a dose of 50 mg TE was added; however, this additional step improved cell viability for the lower dose of 25 mg TE. No statistically significant improvement was observed after the SPE-Florisil step alone was utilized for the NC5 extract.

This approach was also followed to allow the potential evaluation of the same extracts analyzed by LC–MS/MS in order to confirm the toxicity of possible additional toxic compounds initially detected by the mass spectrometer [39,45].

### 2.3. Optimum Maximum Tissue Dose Equivalent (MTDE)

**Figure 3:**
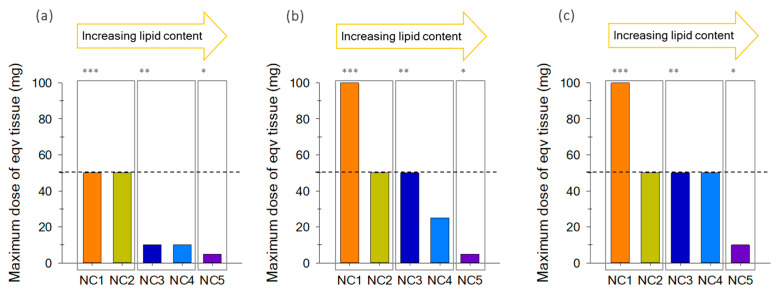
Graph bars shows MTDE for each species, framing * fat fish; ** semi-fat fish; *** low-fat fish. Results were obtained after 16 h exposure to CTX-negative fish extracts obtained by **(a)** Extraction procedure without cleanup step **(b)** SPE Florisil and **(c)** SPE Florisil + SPE-C18 step: *Pagrus pagrus* (orange); *Epinephelus marginatus* (yellow); *Pomatomus saltatrix* (dark blue); *Seriola dumerili* (blue) and *Acantocybium solandri* (purple). Dotted line represents a dose of 50 mg TE.

An alternative method to increase sensitivity while avoiding non-specific toxic effects is the establishment of a MTDE. Therefore, based upon results observed a MTDE was proposed for the different fish species groups (low, semi and fat fish) as well as for steps of purification (no clean-up, SPE Florisil and SPE Florisil+C18; Figure 3).

In the extracts without clean-up, the higher dose of equivalent tissue was established for the low-fat fish NC1 and NC2 (50 mg TE), whereas the lower dose equivalents were obtained for the fish species with the higher lipid content, NC5 (5 mg TE; Figure 3a). Intermediate interference was observed for the semi-fat species, NC3 and NC4, suggesting a MTDE of 10 mg TE.

The addition of Florisil SPE and further C18 steps significantly decreased the non-specific toxic effects for all the fish species, allowing to increase the amount of MTDE and therefore increasing the sensitivity of the assay (Figure 3b,c). For the most purified extracts, the same amount of a tissue equivalent was considered for low-fat and semi-fat fish (50 mg TE). In contrast with the unpurified extracts, the addition of further purification steps, such as SPE Florisil+C18, in order to remove interfering compounds of the matrix implies less dependence between METD and fish lipid content.

The results obtained demonstrated a good correlation between MTDE and lipid content, showing that matrix effects in N2a cells were probably related to interfering factors such as the fatty acids of the different fish species.

The MTDE was established for each type of fish species with different lipid content in order to avoid matrix effects and improve sensitivity. The sample pretreatment followed in this work allows us to propose an amount of 50 mg TE/well (217 mg TE/mL) of wet weight of equivalent tissue for low-fat fish and semi-fat fish as the MTDE. For fat fish 10 mg TE/well (43 mg TE/mL) was proposed.

The limits obtained for low- and semi-fat fish are higher than the proposed in the bibliography (50 mg eqv/mL proposed by Pawlowiez et al. (2013) [41]; A. Caillaud et al. (2012) [35] proposed 20 mg TE/mL to extracts of *Seriola* spp (semi-fat fish) and *Acanthocybium solandri* (fat fish). The results provided by A. Caillaud were obtained according to the protocol described by Lewis et al. (2013), in which no cleanup step was included. A similar result of 5 mg TE/well (22 mg/mL) was obtained in this work for fat fish (*Acanthocybium solandri*) in unpurified extract. The cleanup procedure used in this work, which includes two different SPE steps as described above, allowed increasing this MTDE until 10 mg TE/well (43 mg/mL) was achieved for the same species. Other authors, such as Botein Dechraoui, Tiedeken et al. (2005) [46], reported the use of 20 mg TE/mL in the analysis of a specimen of *Sphyraena barracuda* from Florida using an extraction protocol that includes a SPE silica gel column. The sample extraction procedures used in the different studies (with different purification levels) and the fish species analyzed in each case, could be the reason of the variability of the limits observed in the references cited. Results obtained in this work demonstrated that the variability in the MTDE could be reduced when different species are analyzed including a cleanup procedure, in addition allowing higher assay sensitivity while avoiding matrix effects. However, exposures up to 2000 mg eqv/mL were achieved by Dickey (2008) [38] for more purified fish extracts.

### 2.4. Evaluation of the Toxicity of the Major CTX Analogues

Differences in the toxic potency of the three main CTXs responsible of CFP worldwide, C-CTX1, CTX1B and CTX3C, were evaluated in order to observe the different response in N2a cells. This study is the first time all three congeners were directly compared in the same study. In addition, we evaluated the matrix effect of C-CTX1 and CTX1B in the N2a assay with the objective of minimizing interference and being able to obtain an adequate and reliable semi-quantitative value.

#### 2.4.1. CTX1B, CTX3C and C-CTX1 Toxic Potency in Neat Solution

CTX1B, CTX3C and C-CTX1 toxic potency were evaluated under the same conditions, 16 h of incubation time and a concentration range from 5 to 0.05 pg/well (pg in 10 µL of addition). Dose-response curves were obtained and differences in CTX1B, CTX3C and C-CTX1 toxicity was determined with the inhibitory concentration 50 (IC_50_), which represents the pg of CTXs that produces a reduction of 50% in cell viability.

CTX1B was the most toxic analogue with an IC_50_ of 0.26 ± 0.07 pg/well (*n* = 4), followed by CTX3C with an IC_50_ of 0.03 ± 0.06 pg/well (*n* = 4) and the potency of C-CTX1 was the lowest with an IC_50_ of 0.44 ± 0.07 pg/well (*n* = 7; Table 2).

The three CTXs analogues exhibited a similar toxic response in the N2a assay, in descending toxicity order: CTX1B > CTX3C ≥ CCTX1, where sigmoidal curves fitted showed a slope equal to 2 for all toxins. This is in disagreement with the mouse bioassay (MBA), where CTX1B is ten-fold more toxic than C-CTX1 [38,47]. The use of ouabain and veratridine, in the N2a-MTT assay, which create an artificial environment, and the different expression level of critical ion channels (i.e., VGSCs or NMDA receptors) in N2a cells and mouse target cells could originate the discrepancies observed between N2a-MTT and MBA assay [24,47,48,49,50,51]. Moreover, a possible molecular transformation of the CTXs congeners caused by mouse metabolism or interaction of CTXs with blood could also be considered to explain the discrepancies in the relative toxic potency [24]. However, metabolic pathways for CTXs still need to be established, and the role of an organismal response in contrast to a singular cell line variant in vitro should also be considered.

#### 2.4.2. CTX1B and C-CTX1 Effectiveness in the Presence of the Matrix

The toxin content (ng CTXs g^−1^ fish) in the analysis of different fish species is estimated by comparing the IC_50_ of the CTX pure standard with the IC_50_ of the sample. However, CTXs dose-response curves might be modified in presence of matrix in respect to those obtained from CTXs pure standards, suggesting shifts either in potency (IC_50_-values) or in the effectiveness (slope values). Therefore, the assessment of the matrix influence could avoid an inadequate semi-quantitation of CTXs in naturally contaminated fish samples.

IC_50_ and slope values were calculated according to the dose-response curves using a sigmoid regression curve (4PL) with variable slopes. An estimation of the effect caused by the matrix over the CTXs toxic potency was obtained through the dose-ratio (DR). The DR is defined as the relationship between LD_50_ obtained for CTXs in the presence of the matrix and the IC_50_ obtained for pure CTXs.

On the other hand, the slope determines how the reduction in cell viability occurs as the dose of CTXs is increased. Pöch et al. (1995) [52] proposed a mathematical approach to obtain slope-corrected DR (DR_corr_): log DR_corr_ = (log DR_obs_)∙slope_obs_, where slope_obs_ is the slope coefficient (in absolute value) of the dose-response curve with matrix and DR_obs_ is the DR-value obtained directly from IC_50_s measured in both curves.

DR_corr_ were calculated when significant differences in slope of dose-response curves were determinate. Differences between both slopes and IC_50_-values were evaluated using ANOVA, and applying a Bonferroni´s post hoc test, with a 95% confidence level.

In order to evaluate matrix influence in the CTXs semi quantitation, a standard addition experiment was carried out over non-contaminated fish extracts (*Pagrus pagrus*) without and with clean-up (SPE Florisil+C18). N2a cells were exposed to a fixed amount of matrix ranging from 0.25 to 0.0025 ng CTXs/g fish, using the concentration range of 5–0.05 pg/well. Therefore, in the absence of the matrix effect it would be expected to observe the same slope and IC_50_-values in both cases.

**Figure 4:**
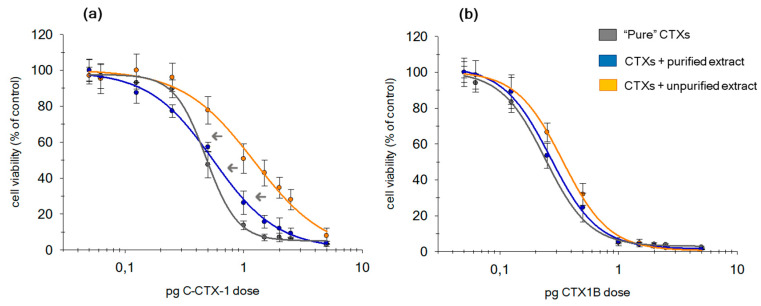
Dose-response curves of N2a cells in +OV conditions (cells with ouabain and veratridine treatment), exposed to an increasing dose of: **(a)** C-CTX1 and **(b)** CTX1B when 20 mg of fish TE, unpurified (orange curves) and purified (blue curves), was added to each well. Gray curves represent response of cells to both CTXs without matrix.

The significant decrease of the C-CTX1 slope in the presence of the matrix is linked with a lower effectiveness (regardless of the level of purification; Figure 4). Therefore, DR-values obtained from those dose-response curves must be corrected using the approach proposed by Pöch et al. (1995) [52]. LD_50_ was corrected using the DR_corr_ according to the relation: IC_50(corr)_ = IC_50(STD pure)_ DR_corr_.

After applying the appropriate corrections, results did not manifest significant differences between C-CTX1 toxic potency when it was analyzed without a matrix and with a purified matrix. However, a significant decrease of the toxic potency of C-CTX1 was observed in the presence of an unpurified matrix, obtaining the lower IC_50(corr)_ (Table 3). In this case, C-CTX1 was 3.6 times less toxic than without a matrix.

Contrarily, CTX1B did not show differences in terms of effectiveness (Table 3). Comparison between toxic potency, with and without matrix, can be estimated with the IC_50_ obtained directly from the curves. CTX1B exhibited almost the same toxic potency without/with a clean-up matrix (Table 3). In the absence of a matrix, a IC_50_ of 0.26 ± 0.02 pg CTX1B/well was obtained while a IC_50_ of 0.27 ± 0.02 pg CTX1B/well, when a cleanup step was applied, was obtained. A significant difference was observed in the IC_50_-values when a cleanup step was not applied, and a little diminution in the toxic potency was observed (1.6 times less toxic).

C-CTX1 and CTX1B effectiveness was clearly different in the presence of matrix. While C-CTX1 was influenced by the matrix reducing the reliability and accuracy of sample semi-quantitation, CTX1B was not affected. These differences might be due to the different affinity of the CTXs to the matrix, which will reduce their affinity to the VGSCs and consequently their effectiveness.

C-CTX1 and CTX1B toxic potency was influenced by the matrix, leading to an overestimation of the toxin content in contaminated samples (Table 3). Therefore, the removal of these compounds by incorporating a cleanup step allowed one to minimize this matrix effect and to obtain a better estimation of both CTXs, especially for CCTX1, which was the most affected.

Limit of detection (LOD) and limit of quantitation (LOQ), corresponding to the concentrations of CTXs necessary to inhibit the cell viability by 10% (IC_90_) and 20% (IC_80_), divided by mg of TE added to the assay (20 mg). The lower sensitivity of the method was obtained in the presence of the unpurified matrix for CCTX1 and CTX1B, but the first one was the most affected. In this case, LODs and LOQs were significantly improved when a clean-up step was included in the sample pretreatment (Table 4). Nevertheless, CTX1B LODs and LOQs were not affected significantly by the elimination of the matrix.

Differences in the toxic effectiveness of both CTXs, in the absence and presence of matrix interfering compounds, was not critical when the N2a-MTT assay was used for screening purposes. On the other hand, significant errors might be obtained when applied to semi-quantitative estimation in samples contaminated with C-CTX1.

## 3. Conclusions

The N2a-MTT assay is considered as a suitable tool to monitor CTX-like compounds, although its applicability in fish samples might be compromised by the matrix effect. A good correlation between MTDE and lipid content of the fish species was observed in this study. The inclusion of a cleanup step by solid phase extraction (SPE) allowed a significant increase of the MTDE for fat fish species and in particular for semi-fat species, the inclusion of this step also contributed to an improved sensitivity. Furthermore, this study also demonstrated that the toxic activity of CCTX1 is highly influenced by the presence of endogenous compounds, suggesting that the matrix compounds might reduce its affinity to the VGSCs. The inclusion of this SPE cleanup will also contribute to a better estimation of naturally incurred C-CTX1 in fish samples therefore increasing the reliability and accuracy of toxin semi-quantitation.

## 4. Materials and Methods

### 4.1. Standards and Reagents

CCTX1 standard solution (10 pg∙µL^−1^) was kindly supplied by Dr. Manger and Dr. Dickey from the Fred Hutchinson Cancer Research Center and the Marine Science institute of the University of Texas (USA), respectively. The CTX1B pure standard solution was kindly provided by professor Takeshi Yasumoto from the Japan food Research Laboratory (JFRL). The CTX3C standard was purchased from Wako Chemicals GmBH (Neuss, Germany).

Acetone, diethyl ether, methanol, water, hexane and ethyl acetate used for extraction and purification were of HPLC grade (Merck KGaA, Darmstadt, Germany). Water for preparation of ouabain and veratridine solutions was deionized and purified at 15 MΩ cm+ through a Milli-Q Gradient A10 system (Millipore, Spain). Water (J. T. Baker, Center Valley, PA, USA) for LC–MS analysis were of LC–MS grade. Methanol (Merck KGaA, Darmstadt, Germany) for diluted samples utilized in the N2a assay were of HPLC grade.

### 4.2. Sample Pretreatment for the N2a-MTT Assay (and HPLC–MS/MS)

Fish samples not containing CTX´s were shipped to the IUSA for testing matrix effects using the N2a-MTT assay. Absence of CTX was confirmed by HPLC–MS/MS. Standard Operating Procedure (SOP) for sample pretreatment was carried out according to conditions initially proposed by Yogui et al. (2011) [53] and modified by Estevez et al. (2019) [39]. Briefly, 15 g of fish tissue were extracted by acetone at 3 mL/g tissue homogenate twice. Acetone was separated from the tissue by centrifugation and dried to an aqueous phase. This phase was extracted with diethyl ether at 1 mL/g fish tissue two times and organic phases were then dried by nitrogen. Residue was partitioned between 90% methanol and n-Hexane (at 0.3 mL 90% MeOH/g fish tissue). The hexane wash was discarded and the methanol phase was dried by nitrogen. Sample cleanup was done with Florisil and C18 solid phase extraction cartridges before analysis by LC–MS/MS. Sample extracts with and without cleanup step were analyzed by an N2a-MTT assay.

### 4.3. In Vitro N2a-MTT Assay

#### 4.3.1. Maintenance of Culture

Neuro-2a (N2a) cell line was obtained from American Type Culture Collection (ATCC, CCL 131, Manassas, VA, USA) and cultured in T75 flask (Corning, NY, USA) in 30 mL of growth medium consisting on RPMI-1640 medium (R8758, Sigma, Irvine, UK) containing 1% (v/v), 100 mM sodium pyruvate (S8636, Sigma, Irvine, UK), 1% (v/v) 200 mM L-glutamine (G7513, Sigma, Irvine, UK) and 1% (v/v) penicillin-streptomycin solution formed by 5000 units and 5 mg/mL, respectively (P4458, Sigma, St. Louis, MO, USA). This medium was supplemented with 10% (v/v) fetal bovine serum (FBS, F2442, Sigma, St. Louis, MO, USA) to obtain a complete growth media (RPMI-10). The N2a cell line was maintained at 37 °C under 5% CO2 in a humidified atmosphere and subcultured when a confluence > 80% was observed (four times per week for a split 1:5).

#### 4.3.2. Cytotoxicity Cell Based assay (N2a-MTT Assay)

The N2a-MTT assay applied to the analysis of fish extracts was developed by Manger et al. (1993) [20] with some modifications. To evaluate the toxic activity of each fraction, prepared from different contaminated samples, cells were seeded into 96-well assay plates (Corning, NY, USA) at a density of 30,000 cells per well in 0.2 mL of growth medium supplemented with 5% (v/v) FBS (RPMI-5) and incubated for 24 h, at 37 °C in humidified atmosphere enriched with 5% CO_2_.

Plates were divided into non-sensitized and sensitized sections. Cells from the sensitized section were exposed to 20 µL/well of a mixture of Ouabain (O3125, Sigma, St. Louis, MO, USA) and Veratridine (V5754, Sigma, St. Louis, MO, USA) (+OV section), prepared from the 10 mM ouabain and 1 mM veratridine stock solutions, in RPMI-C (growth medium without FBS), allowing a reduction of 20% of cell viability. A volume of 20 µL of PBS was added to wells of the non-sensitized cells (-OV section).

Extracts obtained from non-contaminated fish samples in the different steps of the sample pretreatment were evaporated under N_2_ stream at 40 °C until dryness. Then these extracts were reconstituted in MeOH to obtain a concentration of 20 g TE/mL. Non-sensitized cells were exposed to a range of fish extract concentration, which varied from between 0.1 and 100 mg fish TE well^−1^. Serial dilutions were prepared from the extracts of 20 g TE/mL in RPMI-C and 10 µL of each dilution was tested in replicates of 4 wells.

In order to evaluate the matrix effect in the CTXs semi quantification by the N2a-MTT assay, 15 g of a non-contaminated fish sample of *Pagrus pagrus* was extracted. A portion of the sample was collected after extraction and after purification step, then 20 mg TE from each extract was added by well for sensitized and non-sensitized sections by triplicate. Additionally, cells were exposed to CTXs using the concentration range of 5–0.05 pg/well. The CTXs content was equivalent to ranging from 0.25 to 0.0025 ng CTXs/g fish. Dose-response curves were obtained and IC_50_ values calculated.

Sensitivity of the N2a cells to the presence of CTXs compounds was calibrated with a standard solution of C-CTX1, CTX1B and CTX-3C that was serially diluted ten times (ranging from 50 to 0.5 ng mL^−1^). Of each dilution 10 µL was tested in replicates of 4 wells in the presence of OV.

#### 4.3.3. Measurement of Mitochondrial Activity

Following a 16 h incubation with samples, cell viability was assessed by colorimetric MTT (3-(4,5-dimethylthiazol-2-yl)-2,5-diphenyl tetrazolium bromide, Sigma, St. Louis, MO, USA). Briefly, the medium was removed from the wells and replaced by 60 µL of RPMI-5 containing 0.8 mg/mL of MTT. After 40 min at 37°, MTT medium was discarded and the resulting formazan crystals, produced from mitochondrial dehydrogenases of live cells, were solubilized by 100 µL of dimethyl sulfoxide (DMSO, D5879, Honeywell, Seelze, Germany). Absorbance was read with a spectrophotometer (Multiskan^®^ FC Microplate Photometer, Thermo Fisher Scientific Oy, Ratastie, Finland) at 570 nm for testing and 630 nm for reference [54].

#### 4.3.4. Analysis and statistical treatment of the data

Results of cell viability were obtained with SigmaPlot v.12.0 software and were expressed as a percentage relative to control wells. For those data that were fitted to a sigmoidal dose-response curve, the IC_50_ value (concentration of CTXs required to cause 50% reduction in cell viability) was calculated using a four parameters logistic function (4PL) with variable slope. From this equation, the limit of quantitation was calculated by IC_80_ (concentration of CTXs necessary to inhibit the cell viability by 20%). All data are expressed as the means ± SD for n replicates.

## Figures and Tables

**Table 1:** Detailed information of the different fish species studied.

ID Sample	Species	Common Name	Total Lipids* (g/100g)
NC1	*Pagrus pagrus*	Red porgy	<3% (0.7 g/100g)
NC2	*Epinephelus marginatus*	Dusky grouper	<3% (1.0 g/100g)
NC3	*Pomatomus saltatrix*	Bluefish	3–6% (4.2 g/100g)
NC4	*Seriola dumerili*	Greater amberjack	3–6% (5.2 g/100g)
NC5	*Acanthocybium solandri*	Wahoo	>6% (9.4 g/100g)

* Data obtained from National Nutrient Database for Standard Reference (USDA) and F. M. Rueda et al., 2003. These values may change slightly depending on the season, age, sex, size or habitat.

**Table 2:** IC_50_ of the different CTXs analogues evaluated.

CTX Analogue	IC_50_ ± σ (pg·well^−1^)	/Hill Slope/
CTX1B	0.26 ± 0.07	2.3 ± 0.48
CTX3C	0.43 ± 0.06	2.1 ± 0.49
C-CTX1	0.44 ± 0.07	2.4 ± 0.44

**Table 3:** IC_50_ and dose-ratios (DRs)-values of CCTX1 and CTX1B obtained from extracts with and without a cleanup step.

CTXs Type	Analysis Conditions	IC_50_ ± SD (pg∙well^−1^)	ng CTXs∙g^−1^ Fish ± SD	/Hill slope/ ± SD	DR ± SD	DR_corr_ ± SD	IC_50(corr) _ ± SD	ng CTXs∙g^−1^ Fish_corr_ ± SD
**CCTX1**	No matrix	0.48 ± 0.04	0.024 ± 0.002	3.5 ± 0.5	-	-	-	0.024 ± 0.002
	Purified	0.56 ± 0.04	0.027 ± 0.002	1.6 ± 0.1	1.1 ± 0.1	1.3 ± 0.1	0.6 ± 0.1	0.031 ± 0.003
	Unpurified	1.3 ± 0.1	0.06 ± 0.01	1.5 ± 0.3	2.6 ± 0.2	3.6 ± 0.9	1.8 ± 0.2	0.12 ± 0.02
**CTX1B**	No matrix	0.26 ± 0.02	0.013 ± 0.001	2.4 ± 0.1	-	-	-	0.013 ± 0.001
	Purified	0.27 ± 0.02	0.013 ± 0.001	2.4 ± 0.2	1.0 ± 0.1	1.0 ± 0.2	0.27 ± 0.05	0.014 ± 0.003
	Unpurified	0.34 ± 0.02	0.017 ± 0.001	2.3 ± 0.4	1.3 ± 0.1	1.6 ± 0.3	0.4 ± 0.1	0.022 ± 0.006

**Table 4:** LOD and LOQ of CCTX1 and CTX1B.

CTXs Type	Analysis Conditions	LOD		LOQ	
		IC_10_ ± SD (pg∙well^−1^)	ng CTXs∙g^−1^ Fish ± SD	IC_20_ ± SD (pg∙well^−1^)	ng CTXs∙g^−1^ Fish ± SD
**CCTX1**	No matrix	0.20 ± 0.02	0.010 ± 0,002	0.29 ± 0.03	0.014 ± 0.002
	Purified	0.12 ± 0.09	0.008 ± 0,001	0.23 ± 0.02	0.012 ± 0.001
	Unpurified	0.26 ± 0.02	0.013 ± 0,004	0.46 ± 0.09	0.023 ± 0.005
**CTX1B**	No matrix	0.12 ± 0.02	0.007 ± 9 × 10^−3^	0.17 ± 0.02	0.009 ± 9 × 10^−3^
	Purified	0.10 ± 0.01	0.007 ± 6 × 10^−3^	0.14 ± 0.01	0.008 ± 3 × 10^−3^
	Unpurified	0.13 ± 0.01	0.005 ± 5 × 10^−3^	0.19 ± 0.01	0.007 ± 5 × 10^−3^
